# Integrative Analysis of Circadian Transcriptome and Metabolic Network Reveals the Role of De Novo Purine Synthesis in Circadian Control of Cell Cycle

**DOI:** 10.1371/journal.pcbi.1004086

**Published:** 2015-02-25

**Authors:** Ying Li, Guang Li, Benjamin Görling, Burkhard Luy, Jiulin Du, Jun Yan

**Affiliations:** 1 CAS-MPG Partner Institute for Computational Biology, Chinese Academy of Sciences, Shanghai, China; 2 Institute of Organic Chemistry, Karlsruhe Institute of Technology (KIT), Karlsruhe, Germany; 3 Institute for Biological Interfaces, Karlsruhe Institute of Technology, Eggenstein-Leopoldshafen, Karlsruhe, Germany; 4 Institute of Neuroscience, Shanghai Institutes of Biological Sciences, Chinese Academy of Sciences, Shanghai, China; University of Pennsylvania, UNITED STATES

## Abstract

Metabolism is the major output of the circadian clock in many organisms. We developed a computational method to integrate both circadian gene expression and metabolic network. Applying this method to zebrafish circadian transcriptome, we have identified large clusters of metabolic genes containing mostly genes in purine and pyrimidine metabolism in the metabolic network showing similar circadian phases. Our metabolomics analysis found that the level of inosine 5'-monophosphate (IMP), an intermediate metabolite in de novo purine synthesis, showed significant circadian oscillation in larval zebrafish. We focused on IMP dehydrogenase (*impdh*), a rate-limiting enzyme in de novo purine synthesis, with three circadian oscillating gene homologs: *impdh1a*, *impdh1b* and *impdh2*. Functional analysis revealed that *impdh2* contributes to the daily rhythm of S phase in the cell cycle while *impdh1a* contributes to ocular development and pigment synthesis. The three zebrafish homologs of *impdh* are likely regulated by different circadian transcription factors. We propose that the circadian regulation of de novo purine synthesis that supplies crucial building blocks for DNA replication is an important mechanism conferring circadian rhythmicity on the cell cycle. Our method is widely applicable to study the impact of circadian transcriptome on metabolism in complex organisms.

## Author Summary

The circadian timing system gates cell cycle progression in various organisms from unicellular microorganisms to mammals. Although a number of factors have been implicated in linking the circadian clock with the cell cycle, little is known about the contribution of metabolic processes to this interaction. In this study, we have shown that cell cycle, metabolism and the circadian clock are intertwined through consistent circadian expression of genes in de novo purine synthesis in the zebrafish. Three gene homologs of a key enzyme in this pathway, *impdh*, show circadian oscillations in different tissues and have distinct molecular functions. In particular, one of the homologs, *impdh2*, contributes to the daily rhythm of S phase in the cell cycle. We suggest that de novo purine synthesis mediates circadian control of cell cycle, a mechanism that is likely conserved in mammals.

## Introduction

The circadian clock represents a key time-keeping mechanism for many fundamental physiological processes such as cell cycle and metabolism. The cell cycle and the circadian clock are both well-studied periodic processes and their interaction has been a recent focus of circadian research. Until now, it has been observed that the circadian timing system gates cell cycle progression in various organisms from unicellular microorganisms such as *Euglena gracilis* [1–3] and cyanobacteria [4–7] to complex organisms such as humans [8], mouse [9,10] as well as in in vitro culture systems [11]. These findings implicate the cell cycle as being an evolutionarily conserved regulatory target of the circadian clock. The disruption of the circadian timing system leads to cell cycle disorders and subsequently can result in various types of cancers [12–15]. Key circadian clock regulatory targets have been identified as important molecular links between the circadian system and the timing of cell division [9,16–19]. The progression of each phase in the cell cycle is mediated by the activity of cyclin-dependent kinases (CDKs)/Cyclins complexes and CDK inhibitors (CKIs), which are directly or indirectly controlled by key circadian transcriptional factors [19–21]. Additionally, there are substantial lines of evidence suggesting that a number of key cell cycle regulators, such as *p21*, *p16*, *cyclinE*, *cyclinA2*, *wee1*, *cyclinB1*, *cdc2* are rhythmically expressed and clock controlled in many species [9,22,23]. Furthermore, recent studies showed that circadian and cell cycle oscillators were tightly coupled and synchronized in mouse fibroblasts in a 1:1 fashion at the single-cell level [24,25].

Zebrafish represents an excellent vertebrate model to study the interplay between the circadian clock and the cell cycle. Progression through S-phase and M phase is restricted to specific times of the light/dark (LD) cycle in a circadian-dependent manner in zebrafish larvae, adult tissues and even cell lines [22,26,27]. More recently the circadian clock has also been demonstrated to regulate cell proliferation and the expression of core cell cycle regulators in the zebrafish intestine [28]. The detailed mechanism of how the circadian clock orchestrates cell cycle has been studied intensively. Light can serve as an environmental signal that regulates cell division via the circadian clock system [27,29]. Glucocorticoids have also been implicated in playing a key role in the circadian control of cell cycle [30]. Furthermore, ADP, acting as a paracrine signal, has been associated with daily S phase cell activity in adult zebrafish retina [31]. Importantly, the direct clock control of mRNA expression of the cyclin-dependent kinase inhibitors *p20* and *p21* also clearly plays a critical role in cell cycle timing in zebrafish [32].

Recently, there has been considerable interest in how various metabolic processes are controlled by circadian clock [33]. In the meantime, a wealth of circadian transcriptome data has been generated by microarray or RNA-seq studies in different species. Therefore, it is imperative to develop methods to gain insight of the functional consequences of circadian transcriptome on metabolism in the context of global metabolic network. It is well-known that metabolism exerts a strong influence on cell cycle [34]. However, little is known about whether metabolic processes directly contribute to linking the cell cycle and circadian clock. Since the coordination of metabolic pathway activity is an essential prerequisite for major cellular events such as DNA replication during S phase [35,36], we reason that circadian regulation of metabolism may represent a key control point for the cell cycle. In this study, we developed a new clustering method based on a distance measure incorporating both metabolic network and circadian phase information. We applied this method to the circadian transcriptome of zebrafish and systematically identified metabolic gene clusters showing coherent circadian gene expression. We found that the top gene clusters consist of mainly genes in purine and pyrimidine metabolism pathway. In this pathway, Inosine 5'-phosphate dehydrogenase (Impdh) catalyzes NAD^+^-dependent oxidation of inosine 5'-monophosphate (IMP) to xanthosine 5'-monophosphate (XMP), which is the rate-limiting step in the de novo biosynthesis of GTP [37]. Interestingly, three homologous genes of *impdh* in zebrafish, namely *impdh1a*, *impdh1b* and *impdh2*, all show robust circadian expression in both larval and adult zebrafish but reside in two different coherent circadian gene clusters. Consistent to this finding, we observed that the level of IMP also showed circadian oscillation in zebrafish larvae through metabolomics analysis. We showed that while *impdh1a* contributes to eye development and the synthesis and distribution of pigment, *impdh2* plays an important role in the circadian control of cell cycle. Furthermore, *impdh1b* function serves to delay embryonic development, contrary to *impdh2* function. The pharmacological inhibition of Impdh activity by mycophenolic acid (MPA) treatment attenuates the circadian control of cell cycle. Gene-specific knock down experiments revealed that the inhibition of *impdh2* function is mainly responsible for this phenomenon. Thus, we hypothesize that purine synthesis is circadian clock-controlled via *impdh* activity and contributes to the daily rhythm of S phase in the cell cycle. Finally, in the light of findings from the mouse, we proposed that de novo purine synthesis may also mediate circadian rhythm of cell cycle in mammals. Taken together, our study suggests that circadian control of de novo purine synthesis represents a novel mechanism linking the circadian clock, metabolism and the cell cycle.

## Results

### Coherent circadian expression in zebrafish metabolism

Our previous study has revealed that zebrafish start to show robust circadian activity in the larval stage at 5 days post-fertilization (5 dpf) [38]. We have identified 2,847 zebrafish circadian oscillating genes (ZCOGs) under both LD and DD conditions in larval zebrafish data. In this study, we focus on the circadian regulation of metabolism. Due to a lack of a global metabolic network in zebrafish as in the case of human and mouse [39], we compiled a zebrafish metabolic network from the information of metabolic reactions, enzymes, and genes of zebrafish from KEGG database [40,41] (S1 Dataset). Such network provides global connectivity between enzymes or reactions through metabolites in an unbiased fashion. Considering one reaction usually contains multiple genes and one gene may be involved in multiple reactions, we combined both gene symbol and KEGG reaction ID to uniquely represent each reaction in the network. We mapped all ZCOGs in larval zebrafish onto zebrafish metabolic network. We obtained 317 ZCOGs coding for enzymes catalyzing 1034 reactions in total. These circadian oscillating enzymes are overrepresented among ZCOGs (p<0.0001, Fisher’s exact test). As circadian phase of ZCOG can be used as the proxy for the peak of circadian activity of enzymes, we defined the distance measure *D* between reactions incorporating the phase information of ZCOGs together with the proximities of the reactions on the zebrafish metabolic network:

$$D_{ij}=d_{ij}+4\lambda \sin^{2}\left(\frac{p_{i}-p_{j}}{24}\right)\pi$$

**Fig 1:**
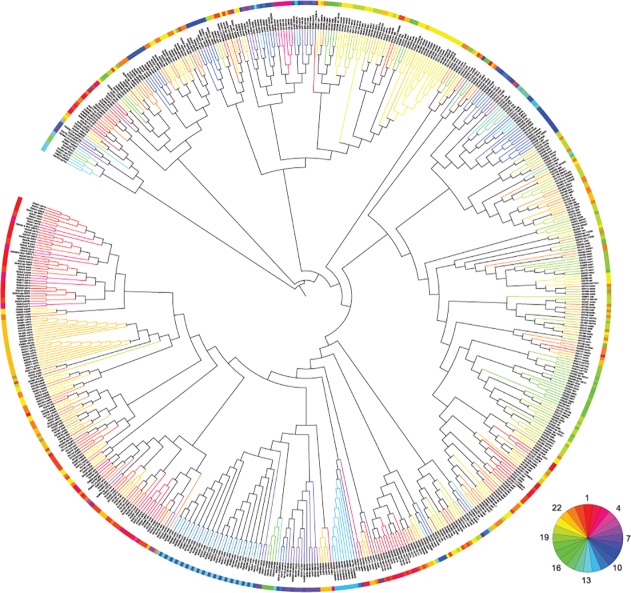
The clustering of ZCOGs on zebrafish metabolic network. Larval ZCOGs and metabolic reactions from KEGG were clustered based on zebrafish metabolic network and circadian phases. Both gene symbol and KEGG reaction ID are used to represent gene and reaction. The tree was visualized using iTOL [42,43]. The colors on the outer ring and tree branches represent the circadian phases of ZCOGs according to the pie chart in the bottom right. The main cluster is shown here. The rest of smaller isolated clusters can be found in S1 Fig.

Here *d_ij_* is the shortest distance between the corresponding reactions *i* and *j* on the network with circadian phases *p_i_* and *p_j_* respectively. *λ* is a positive parameter to adjust the contribution from phase difference. We then applied a hierarchical clustering of reactions based on these distances. We obtained a main cluster of 632 reactions together with 30 other isolated clusters of much smaller sizes. The tree structure of the main cluster is shown in Fig. 1 when *λ* = 1. Five smaller isolated clusters with size larger than 10 are shown in S1 Fig. We defined coherent circadian gene clusters as the clusters obtained from truncating the tree at height with *h* = 12 so that the genes in each cluster showed coherent circadian phases. The summary of top 10 coherent circadian gene clusters is shown in Table 1. The genes in the same cluster tend to belong to the closely related metabolic pathways. In particular, we found the top three clusters are all enriched with genes in purine/pyrimidine metabolism. Furthermore, these three clusters showed coherent circadian gene expression around CT2 (circadian time 2), CT4, and CT15 respectively, where CT0 is subjective lights-on. Among them, we found that three *impdh* gene homologs, *impdh1a*, *impdh1b* and *impdh2* showing strong circadian rhythms of expression fall into two different coherent circadian gene clusters. The peaks of rhythmic expression of *impdh2* and *impdh1a* were observed at CT23 in cluster 1, while *impdh1b* exhibited peak expression at around CT12 in cluster 3. In summary, our method segregated ZCOGs in both metabolic pathways and circadian phases.

**Table 1:** Summary of top 10 coherent circadian gene clusters in zebrafish metabolic network.

Cluster ID	Mean circadian phase (CT)	Coherency in circadian phases (*p* value)	Number of genes per reactions	Enriched KEGG pathway
1	1.8	5.98×10^−46^	145	Pyrimidine metabolism, Purine metabolism
2	4.8	1.00×10^−41^	121	Purine metabolism, Fatty acid degradation
3	14.5	1.45×10^−22^	58	Pyrimidine metabolism, Purine metabolism
4	4.9	3.19×10^−22^	53	Arachidonic acid metabolism, Glycerophospholipid metabolism
5	15.4	2.75×10^−05^	31	Nicotinate and nicotinamide metabolism
6	4.6	1.56×10^−10^	31	Pentose phosphate pathway, Starch and sucrose metabolism
7	8.9	1.78×10^−03^	31	Metabolism of xenobiotics by cytochrome P450
8	17.8	1.07×10^−03^	29	Pyrimidine metabolism, Purine metabolism
9	8.7	7.62×10^−03^	26	Metabolism of xenobiotics by cytochrome P450
10	3.3	1.21×10^−03^	23	Tyrosine metabolism

### Circadian metabolome in larval zebrafish

**Fig 2:**
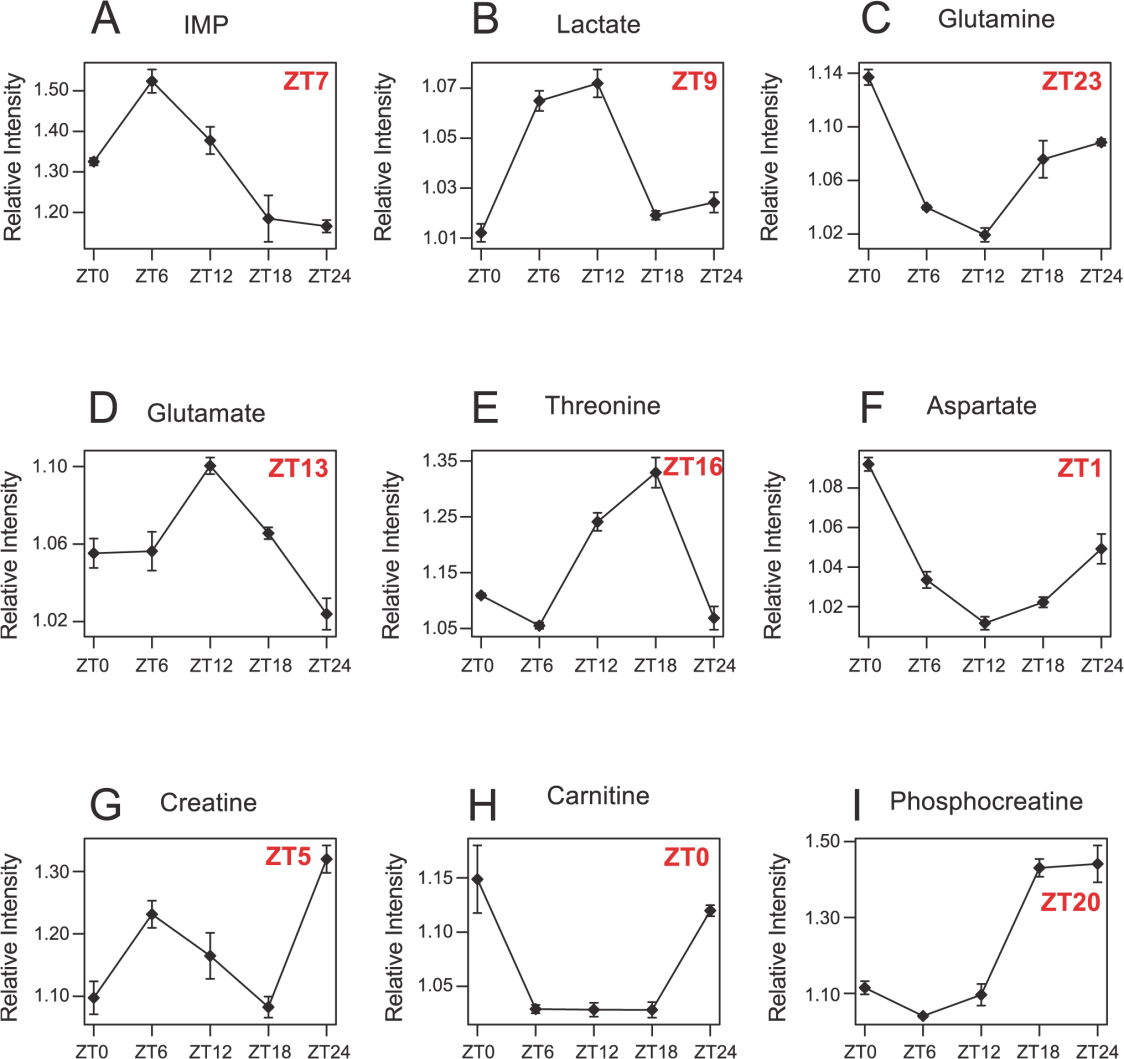
Circadian metabolites in larval zebrafish. The quantification of nine circadian metabolites including IMP (A), lactate (B), glutamine (C), glutamate (D), threonine (E), aspartate (F), creatine (G), carnitine (H), and phosphocreatine (I) by NMR. The y-axis is scaled intensity in NMR measurement relative to the sample with the lowest intensity for each metabolite. The circadian phases of metabolites are also shown. Error bars represent the standard error of mean (SEM) among independent biological replicates at each time point.

We next measured the levels of water-soluble metabolites in 5dpf larval zebrafish collected every six hours during 24 hours (ZT0-ZT24) under LD condition using a hydrogen nuclear magnetic resonance (^1^H NMR) spectroscopy. A total of 173 peak signals were quantified based on their spectral intensities. 73 of them can be assigned to known metabolites including amino acids and nucleotides (S1 Table). We found that 18 of them showed significant circadian oscillation (cosine fitting p value<0.05 and ANOVA p value<0.05). These correspond to nine unique known metabolites as shown in Fig. 2. IMP showed robust circadian rhythm with its peak time around ZT7. The rest of circadian metabolites include lactate (ZT9), glutamine (ZT23), glutamate (ZT13), threonine (ZT16), aspartate (ZT1), creatine (ZT5), carnitine (ZT0), and phosphocreatine (ZT20). We further identified ZCOGs that are linked to these circadian metabolites on our zebrafish metabolic network (S2 Table). Lactate is connected with lactate dehydrogenase Bb (ldhbb) peaking at CT17. Creatine and phosphocreatine are connected with creatine kinase brain b (ckbb) peaking at CT4. Glutamine and glutamate are connected with glutamine synthase, glula and glulb, peaking at CT5 and CT6 respectively. In particular, IMP is directly connected with ten circadian enzymes including impdh homologs in de novo purine synthesis pathway. Therefore, the circadian oscillation of IMP further supports the circadian rhythm of de novo purine synthesis from their enzyme expression.

### Circadian expression rhythms of three *impdh* gene homologs in adult zebrafish

We next investigated the circadian gene expression in adult zebrafish. We monitored the circadian behavior of adult zebrafish (6 months old males) under an infrared behavioral monitoring platform specially designed for adult zebrafish (S2A Fig.). Wild-type (WT) zebrafish were raised in 14h:10h light/dark (LD) cycle from birth. Adult zebrafish showed robust circadian changes in their locomotor activities under LD (S2B Fig.) while the amplitude of oscillation was significantly lower in dark/dark (DD) conditions (S2C Fig.). Such strong dependence of circadian activities on light has also been seen in larval zebrafish [38]. The circadian activity in adult zebrafish is more robust than that in larval zebrafish in terms of the baseline activity and the amplitude of the rhythm.

**Fig 3:**
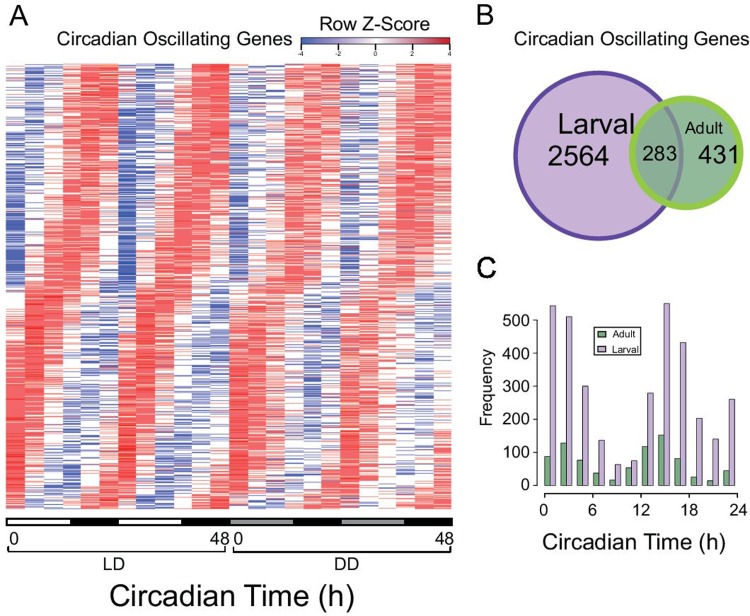
Global circadian gene expression in adult zebrafish brain. (A) Circadian expression of adult ZCOGs under both LD and DD conditions. High expression is indicated in red and low expression is in blue. The bar on the x-axis indicates light (white) and dark (black) in LD, and subjective day (gray) and subjective night (black) in DD. (B) Venn diagram quantifying the intersection of ZCOGs in adult (green) and larval (purple) zebrafish. (C) Bimodal distribution of circadian phases of ZCOGs in adult (green) and larval (purple) zebrafish.

**Fig 4:**
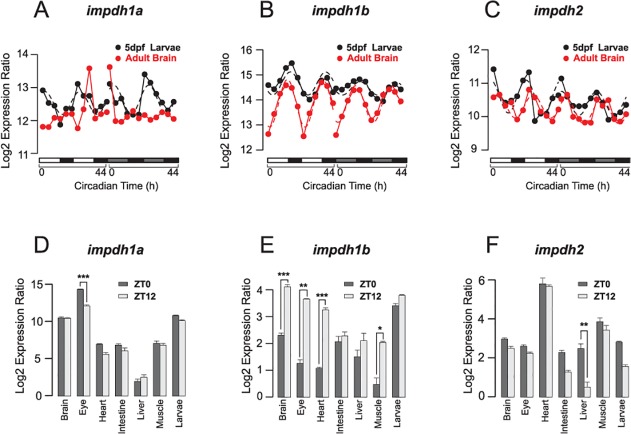
The circadian expression of three *impdh* homologs show robust in larvae and adult zebrafish tissues. The expression of *impdh1a* (A) is not circadian oscillating in adult zebrafish brain (red line) but shows circadian rhythm in 5dpf larval whole body (black line). *impdh1b* (B) and *impdh2* (C) are circadian oscillating with circadian phases around CT12 and CT23 respectively in both adult zebrafish brain and larvae. The solid curves represent microarray data and the dashed curves were fitted by cosine functions. (D) *impdh1a* is predominantly expressed in the adult eye where the expression level at ZT0 is much higher than that at ZT12. (E) *impdh1b* shows rhythmic expression in the brain, eye, heart and muscle. (F) Although the expression of *impdh2* in the heart is higher than in other tissues, it shows the most significant expression change between ZT0 and ZT12 in the liver. Error bars represent the standard error of mean (SEM) among independent biological replicates. The gene expression levels at ZT0 and ZT12 were compared using a Student’s t-test (unpaired, two tailed; *** p<0.001; ** p<0.01; * p<0.05).

We next collected adult zebrafish whole brain every four hours during 48 hours under both LD and DD conditions. The locomotor activity of each adult fish was recorded before being sacrificed (S3 Fig.). The whole-genome transcriptome profiles of adult brain in this time-series were assayed using Agilent zebrafish microarrays. We used a similar statistical method to identify zebrafish circadian genes (ZCOG) as our previous study in larval zebrafish. Under a False Discovery Rate (FDR) <0.05, we identified 714 ZCOGs in adult brain under both LD and DD conditions (Fig. 3A). 283 ZCOGs were shared by both adult brain and larval ZCOGs (S2 Dataset and Fig. 3B) with many exhibiting a similar phase of rhythmic expression during zebrafish post embryonic development (Fig. 3C). In adult brain, *impdh1b* and *impdh2* again showed strong circadian rhythms of expression with *impdh1b* expression peaking at CT12, and *impdh2* peaking at around CT23 as in larvae. However, *impdh1a* did not show significant rhythm in adult brain (Fig. 4A, 4B, 4C).

We next collected five additional tissues (eye, intestine, liver, heart and muscle in addition to whole brain) from adult fish maintained under LD cycles at zeitgeber time 0 (ZT0) (where ZT0 is defined as lights-on and ZT14 is defined as lights-off) and ZT12. Using real-time PCR, we revealed that *impdh1a* was predominantly expressed in the eye, where the expression level at ZT0 was considerably higher than at ZT12 (Fig. 4D). *impdh1b* exhibited expression in all tissues, with higher expression at ZT12 than ZT0 in each case (Fig. 4E). Although the expression levels of *impdh2* in the heart, muscle and liver were much higher compared with other tissues, the most significant circadian oscillation appeared in the liver (Fig. 4F). These results validated and extended our microarray results. Thus, the circadian expression of three *impdh* gene homologs are conserved between larval and adult zebrafish. From their spatial and temporal expression patterns, we hypothesize that the three *impdh* gene homologs have different tissue-specific functions. While *impdh1b* may play a “housekeeping” role in most tissues, *impdh1a* is likely to have an eye-related function and *impdh2* may function in metabolically active tissues.

### Distinct functions of the three *impdh* homologs in early development

**Fig 5:**
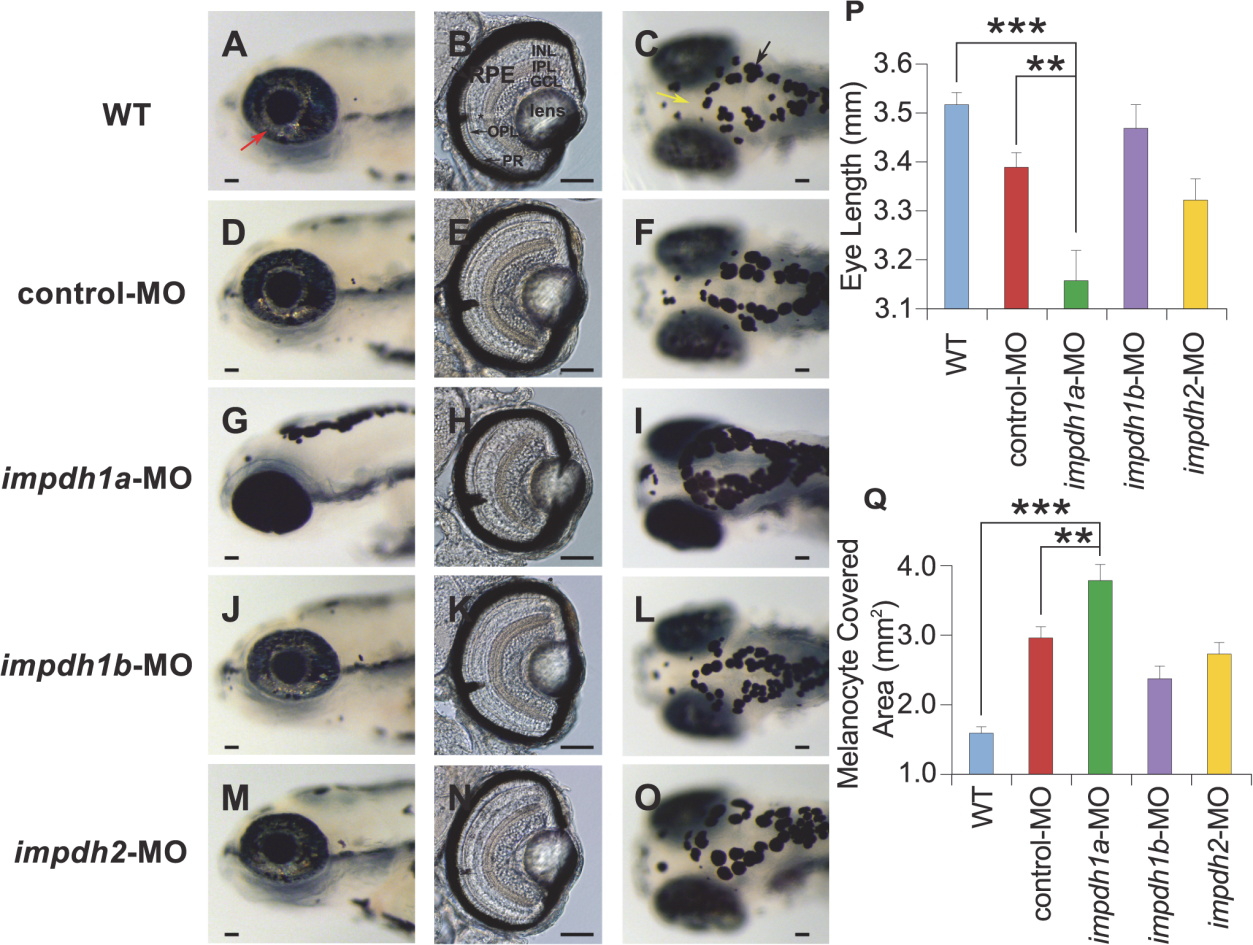
*impdh1a* knock-down leads to pigmentation defects and smaller eyes. (A,D,G,J,M): Coronal planes through 5 dpf larval eyes. Xanthophores (yellow arrow) and iridophores (red arrow) were reduced significantly in *impdh1a* morphants. (B,E,H,K,N): Transverse histological sections of *impdh* morphants. The eye length along the dorsal/ventral axis was much shorter in *impdh1a* morphants. There was no apparent phenotype in retinal cell types between WT, control and the three *impdh* morphants; (C,F,I,L,O): Dorsal images of 5 dpf larvae showed that melanophores (black arrow) covered a larger area of the head in *impdh1a* morphants. Anterior-posterior eye length (P) and melanocyte covered area (Q) were measured and compared using a Student’s t-test (unpaired, two tailed; *** p<0.001; ** p<0.01; * p<0.05). At least ten larvae were tested in each group. Error bars represent the standard error of mean (SEM) among independent biological replicates. GCL: ganglion cell layer, INL: inner nuclear layer, IPL: inner plexiform layer, OPL, outer plexiform layer, PR: photoreceptor layer, RPE: retinal pigment epithelium. Scale bars, 50μm.

To explore the functions of the three *impdh* gene homologs, we knocked down *impdh1a*, *impdh1b* and *impdh2* expression using morpholino oligonucleotides (MOs) respectively. The phenotypes of the three *impdh* morphants (MO injected larvae) at 5 dpf are shown in Fig. 5A-5O. WT zebrafish possess three types of pigment: silver or reflective pigment produced by iridophores (red arrow in Fig. 5A), yellow pigment derived from xanthophores (yellow arrow in Fig. 5C) and black pigment derived from melanophores such as that in the retinal pigment epithelium (RPE) (black arrow in Fig. 5C). In WT zebrafish embryos, black pigment begins to form around the Prim-5 stage (24 hpf), xanthophores first appear on the fish head and iridophores are first present on the fish eye at the long-pec stage (48 hpf) [44]. When *impdh1a* was knocked down, pigmentation defects were visible and easily recognized starting from 72 hpf. At 5 dpf in WT embryos, the iridophore retinal ring is normally filled out and iridophores are densely packed in a dorsal stripe. In contrast, nearly all yellow and silver pigments were absent in *impdh1a* morphants (Fig. 5G, I) and the area covered by melanocytes in the head region was much broader in *impdh1a* morphants than those in other *impdh* morphants and controls (Fig. 5C, F, I, L, O, and Q for quantification).

In addition to loss of pigment, *impdh1a* knock-down specifically led to microphthalmia, suggesting that *impdh1a* plays a crucial role in regulating eye growth. As shown in Fig. 5P, the retina size measured along the anterior to posterior axis was much smaller in *impdh1a* morphants than in the control groups. Therefore we dissected and sectioned the eyes into slices along the transverse axis to examine in more detail the eye anatomy. In histological imaging of the retina (Fig. 5B, E, H, K, N), none of three *impdh* homologs morphants showed alterations in the retinal cell types. Furthermore, there were no obvious differences in lens area or pigmentation of the RPE between *impdh1a*, *impdh1b*, *impdh2* morphants, control and WT except that the overall eye size was much smaller in the *impdh1a* morphants than in controls. In contrast, both *impdh1b* and *impdh2* morphants displayed normal eye morphology and pigmentation (Fig. 5).

Interestingly, the rate of growth of the *impdh1b* morphants was higher than controls while *impdh2* morphants developed more slowly. Thus at 32 hpf, *impdh2* morphants showed a 6–8 h developmental delay (S4A Fig.) and only hatched at 52 hpf. In contrast, the more rapid growth of *impdh1b* morphants was visualized by their longer body length measured between 52 hpf and 5dpf (S4A, B Fig.). Therefore, our data suggest that while *impdh2* function tends to increase the rate of zebrafish embryonic development, *impdh1b* inhibits early growth.

### *impdh2* mediates the circadian control of cell cycle

**Fig 6:**
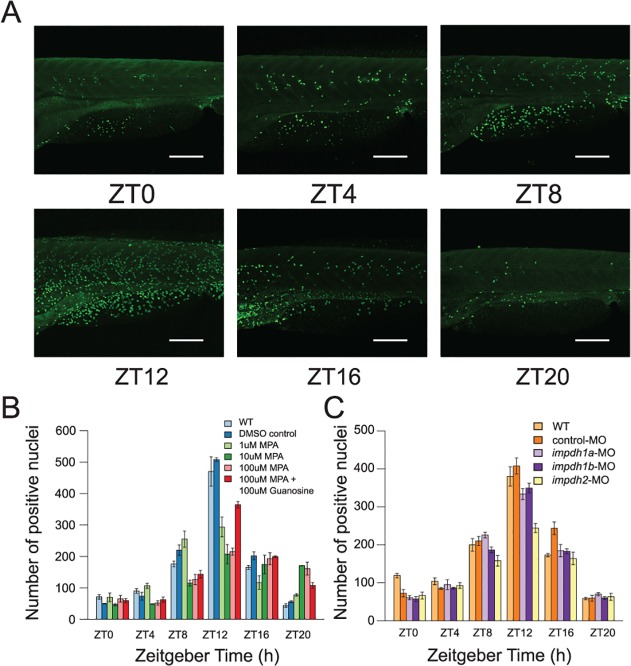
*impdh2* mediates the circadian control of cell cycle. (A) The BrdU staining in 5 dpf larvae represents the daily rhythm of S phase in cell cycle under LD conditions. (B) The amplitude of the S phase rhythm was attenuated when the larvae were treated with MPA. The effect of MPA was rescued when guanosine was added to MPA-treated larvae. The inhibitory effect of MPA on the S phase rhythm was in a dose-dependent manner and saturated at 10μM concentration. (C) The amplitude of the S phase rhythm decreased significantly in *impdh2* morphants. At least three larvae were measured at each data point. Error bars represent the standard error of mean (SEM) among independent biological replicates. Scale bars, 200μm.

Impdh is well known as a rate-limiting enzyme in the de novo biosynthesis of guanine nucleotides. As purines are basic building blocks of DNA and RNA that are needed in cell proliferation, we reason that the circadian rhythm of *impdh* may influence the rhythm of cell entry into S phase rhythm. To test our hypothesis, we first used MPA, a selective inhibitor of *impdh* protein, to treat larval zebrafish. We used BrdU to label S-phase nuclei in larvae at six circadian time points as shown in Fig. 6A. In 5 dpf WT larvae, we counted the number of Brdu positive nuclei and found a strong daily S-phase rhythm across 11 somites of the fish trunk (Fig. 6B). We then treated 4 dpf larval zebrafish with MPA at different concentrations and sampled fish starting at ZT0 of 5 dpf at 4h intervals. Larval zebrafish did not show altered morphology or behavioral changes when MPA concentration was below 100μM. MPA treatment significantly reduced the amplitude of the S phase rhythm in a dose dependent manner. This reduction in rhythmic cell proliferation was rescued when 100μM guanosine was added to MPA-treated larval fish (Fig. 6B). These results suggest that MPA inhibits circadian rhythmicity of S phase by limiting the GTP required by DNA synthesis and that de novo purine synthesis plays an important role on the circadian rhythmicity of cell cycle.

Next, in order to examine which *impdh* gene homolog actually contributes to circadian control of cell cycle, we knocked down the three *impdh* homologs individually using MOs and studied the effect on the circadian rhythm of S phase. We failed to observe any effect of *impdh1a* MO or *impdh1b* MO on cell cycle rhythmicity. In contrast, the high-amplitude daily rhythm of S phase was significantly suppressed when *impdh2* was knocked down (Fig. 6C). This result suggested that *impdh2* plays a major role in circadian control of cell cycle.

### Genome-wide effects of *impdh* homolog-specific knock-downs

Impdh has been well documented to serve as a key enzyme in the GTP biosynthesis pathway. In order to explore in more detail the mechanisms whereby changes in purine biosynthesis might generate the overt aspects of the morphant phenotypes, we measured global transcriptome changes in the whole body of *impdh1a*, *impdh1b* and *impdh2* morphants (32hpf) using RNA-seq. We identified 468, 331 and 1166 genes whose expression was significantly altered in the *impdh1a*, *impdh1b* and *impdh2* MO knock-down compared to controls (S5A Fig.). There was only limited overlap amongst these three groups, with only 36 genes showing altered expression in all three MOs (S5B Fig.). This indicates that the down-regulations of three *impdh* homologs have distinct impacts on global gene expression.

We next classified the genes affected by *impdh*-specific knock-downs into KEGG pathways and examined their tissue specific expression patterns based on annotations in the ZFIN database [38]. The expression of genes affected by the *impdh1a* knock-down is enriched in retina inner/outer nuclear layers (S3 Dataset) while those affected by *impdh1b* and *impdh2* knock-downs are enriched in the regulation of developmental process. Thus, *rpe65a* involved in visual pigment regeneration and *ddt* in melanin synthesis are significantly up-regulated upon *impdh1a* knock-down. Both *rpe65a* and *ddt* display circadian rhythms of expression with peaks at CT24 and CT7 respectively. Interestingly, mutations in RPE65 cause retinal pigmentosa similarly to IMPDH1 mutations [45]. *dhfr* which synthesizes tetrahydrofolate essential for purine metabolism is down-regulated in both *impdh1a* and *impdh1b* morphants. In addition, metabolic enzymes including *psat1* and *phgdh* involved in serine synthesis and *bcat1* in the synthesis of branched chain amino acids were up-regulated in *impdh2* morphants. Both serine and branched chain amino acid metabolism have been implicated as key requirements for cell proliferation [46,47]. The differential expression of *rpe65a*, *dhfr*, *bcat1* and *psat1* were all validated by real-time PCR (S6 Fig., primers used for real-time PCR are in S3 Table). Thus, the molecular functions of the genes affected by three *impdh* homolog-specific knock-downs provided the clues to the molecular basis of the morphant phenotypes that we observed.

### Circadian regulation of de novo purine synthesis pathway

**Fig 7:**
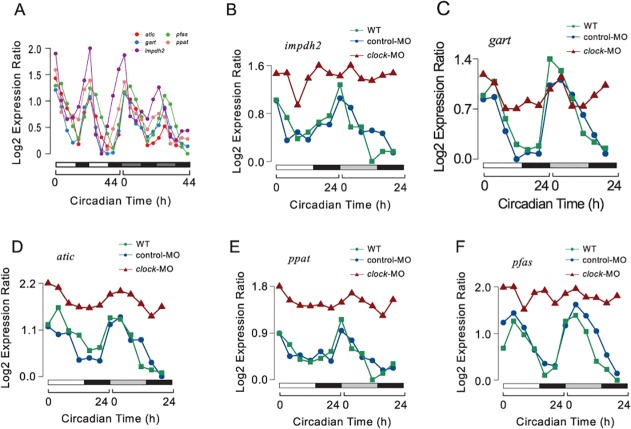
Circadian clock controls de novo purine synthesis pathway. (A) Circadian oscillating genes in de novo purine synthesis pathway have similar circadian phases (around CT0) in both LD and DD conditions. (B-F) *clock* controls the circadian expression of these genes as indicated by the significantly dampened oscillations in *clock* morphants (red) compared to WT (green) or control morphants (blue) in both LD and DD. The lowest log2-transformed expression level for each gene was normalized to zero.

In our larval zebrafish microarray data, the expression of genes encoding multiple enzymes upstream of *impdh* in de novo purine synthesis, including *atic*, *gart*, *pfas*, *ppat* together with *impdh2*, all displayed similar circadian rhythms (Fig. 7A). We further studied their expression in six adult tissues at ZT0 and ZT12 using real-time PCR as shown in S7 Fig. (primers used for real-time PCR are in S3 Table). Similar to *impdh2*, all genes showed elevated expression at ZT0 in liver and other tissues. To investigate whether these genes in the purine synthesis pathway were regulated by the core circadian clock mechanism, we generated *clock* MO knock-down larvae. Time-series measurements of *atic*, *gart*, *pfas*, *ppat* and *impdh2* mRNA levels by real-time PCR showed significantly dampened oscillations in 5 dpf *clock* morphants compared to WT or control morphants in both LD and DD conditions (Fig. 7B-7F). Thus, these circadian oscillating genes in de novo purine synthesis are likely to be co-regulated by *clock*. We examined a published *Bmal1* ChIP-seq data in mouse liver [48]. In mouse, *Ppat* and *Paics* share the same promoter, which contains a strong *Bmal1* binding site. *Atic* also has a *Bmal1* binding site in its promoter. We conducted promoter analysis for the corresponding zebrafish genes. We identified a Bmal1/Clock binding site in the promoter of *ppat*, *paics*, and *impdh2* in zebrafish strongly suggesting that they are also under the direct control of Bmal1/Clock.

In mouse retina, ChIP-seq analysis has revealed that *Impdh1* is regulated by *Crx*, a transcription factor specifically expressed in the eye and pineal gland [49]. In our study, *crx* showed circadian expression peaking at CT17 preceding the peak of *impdh1a*. The circadian oscillation of *impdh1a* may be directly regulated by *crx* and indirectly driven by *clock*. Our functional analysis implicates *impdh1a* in melanogenesis and eye development. Our previous study has shown that melanogenesis exhibits a circadian rhythm with a peak around CT0 similar to rhythmic *impdh1a* expression. Therefore, the circadian oscillation of *impdh1a* may contribute to circadian rhythm of melanogenesis in zebrafish. In the promoter of *impdh1b*, we encountered a retinoic acid-related orphan receptor (ROR) response element (RRE), the binding site for ROR and NR1D family transcription factors. This result is consistent with the CT12 peak of rhythmic *impdh1b* expression since we observed previously that circadian oscillating genes with peaks around CT12 tend to contain RRE elements [38]. Taken together, we proposed that the three zebrafish homologs of *impdh* are regulated by different circadian transcription factors which lead to their different circadian phases and tissue distribution.

## Discussion

Previously, we have demonstrated that a functional circadian clock is already present at 5 dpf in larval zebrafish and we have identified more than 2,800 ZCOGs using whole larval RNA extracts and a high-throughput approach [38]. To examine the effects of these ZCOGs on metabolism, we developed a computational method to partition different metabolic processes according to time of day. Our clustering method is based on two assumptions. First, the enzymes similar in circadian phases of gene expression at the transcriptome level also tend to reach their activity peaks approximately at the same time of day. Second, the co-expressed circadian enzymes that are proximal in metabolic network form a module of common metabolic function. Under these assumptions, our method should be generally applicable to the integration of circadian transcriptome data and global metabolic network. Applying our method to zebrafish circadian transcriptome, we found that top clusters of ZCOGs with coherent circadian expression are enriched with genes in purine and pyrimidine metabolism. At a rate-limiting step of this pathway, three *impdh* gene homologs that have not been previously implicated in circadian clock function, all showed robust, high amplitude circadian rhythms. Furthermore, their circadian expression is developmentally conserved between larval and adult zebrafish. Subsequent functional analysis of these *impdh* homologs revealed that *impdh1a* is involved in pigment synthesis and eye development while *impdh2* is generally implicated in the control of cell proliferation. Expression of both *impdh1a* and *impdh2* oscillated with circadian peaks at CT23. In comparison, *impdh1b* was ubiquitously expressed and showed an opposite phase of rhythmic expression to *impdh1a* and *impdh2* in all tissues tested. Although the detailed functions of *impdh1b* are still unclear, it seems that it may have the function to delay growth in contrast to both *impdh1a* and *impdh2* which appear to accelerate growth. Our RNA-seq analysis of *impdh* homolog-specific morphants identified downstream genes that may be responsible for the different phenotypes. Our promoter analysis also suggested that the rhythmic expression of the three *impdh* homologs was controlled by different circadian transcription factors: *impdh2* was regulated by Bmal1/Clock, *impdh1a* by Crx, and *impdh1b* by Ror or Nr1d.

Fustin et. al (2012) have reported the circadian expression of genes involved in purine and pyrimidine nucleotide metabolism in mouse liver [50]. They revealed that circadian rhythms of *Ppat* and *Pnp* expression contribute to rhythmic synthesis of guanine nucleotides but they fell short of linking this phenomenon to circadian control of the cell cycle. In mouse, *Ppat* shows strong circadian oscillation in multiple tissues with a peak at CT11, typical for Bmal1/Clock regulated circadian genes. Consistently, our previous larval study identified rhythmic expression of several *pnp* homologs in zebrafish [38]. Our results showed that both MPA inhibition and impdh2 knockdown have significantly dampened the amplitude of circadian rhythm of S phase in proliferating cells of zebrafish. This is consistent with the earlier report that MPA and impdh2 have the same inhibitory effect on the sprouting of intersegmental blood vessels in early zebrafish larvae [51]. We found that IMP, the substrate for Impdh2, is peaking at ZT7, five hours ahead of the peak of S phase. This observation suggests that the peak of Impdh2 activity may be between ZT7 and ZT12 to deplete IMP level to provide purine for DNA synthesis. Thus the circadian oscillation of IMP together with *impdh2* can have an important regulatory role on the metabolic flux through purine synthesis pathway. From the microarray data for mouse tissues and cell lines in the BioGPS database (http://biogps.org/), mouse *Impdh2* together with other de novo purine synthesis genes are highly expressed in embryonic stem cells. From the in situ data for the developing mouse embryo at E14.5 in Metscout database (http://www.metscout.mpg.de), *Impdh2* is expressed in proliferating cells including those in the ventricular zone of the brain similar to other enzymes involved in de novo purine synthesis. It was shown that enzymes in de novo purine synthesis form reversible multi-enzyme complex in so-called “purinosome” in HeLa cells [52]. These observations suggest that the enzymes in de novo purine synthesis pathway are co-regulated both spatially and temporally to control metabolic flux. Karpowicz et al. (2013) have found that the circadian clock regulates stem cell regeneration in the fly intestine [53]. In mammals, there are also reports of circadian clock regulation in epidermal cells [54] and hematopoietic stem cells [55]. Therefore, our results suggest that circadian purine synthesis may directly contribute to cell proliferation and stem cell regeneration regulation by the circadian clock in a highly conserved manner.

Our study has revealed that *impdh1a* is required for pigment synthesis and ocular development. Interestingly, the *gart* and *paics* mutants which affect two enzymes upstream of Impdh in de novo purine synthesis, display almost identical phenotypes in the zebrafish retina as our *impdh1a* morphant [56]. From this previous study it was proposed that purine synthesis provides key precursors for three types of pigment cells in zebrafish: iridophores, xanthophores and melanophores [56]. Our results suggest that *impdh1a* is also involved in these processes. This is consistent with our previous finding that melanogenesis is under circadian clock control in zebrafish [38]. Therefore, it seems that *impdh1a* and *impdh2* along with other enzymes in de novo purine synthesis are involved in circadian regulation of two seemingly different processes that are both controlled by purine synthesis: pigment synthesis in pigment cells and cell growth in proliferating cells. This functional difference is also revealed by the distinct differential gene expression upon their MO knockdowns.

It is well known that circadian clock dysfunction is closely associated with metabolic disorders or diseases. Metabolism-based research in circadian rhythms represents a potentially important contribution to our understanding of circadian output functions [57]. The mis-regulation of enzymes in nucleotide metabolism is especially prone to diseases. Only two IMPDH homologs, IMPDH1 and IMPDH2, exist in humans sharing 84% amino acid identity [58]. The amino acid sequences of Impdh homologs are highly conserved between human, mouse and zebrafish [51]. Thus, zebrafish Impdh1a shares 90% identity and Impdh1b, 91% identity with human IMPDH1 (S8 Fig.). In addition, zebrafish Impdh2 shares 91% identity with human IMPDH2. Among the two IMPDH homologs that have been described in humans, IMPDH1 is linked to several types of severe retinal degeneration including retinitis pigmentosa and Leber congenital amaurosis (LCA) [45]. Furthermore, IMPDH2 contributes to cell proliferation in a variety of cell lines and tissues such as activated lymphocytes and tumor cells [59–61]. Thus, IMPDH has already become an important drug target for antiviral, cancer chemotherapy and immunosuppressive therapy. Immediately downstream of IMPDH, hypoxanthine phosphoribosyltransferase (HPRT) that also showed circadian expression similar to IMPDH2 in our study is implicated in Lesch-Nyhan syndrome, a neurological disease [62]. Therefore, our study provides novel insight into whether enzymes in de novo purine synthesis, especially IMPDH, can be used as biomarkers for metabolic diseases which are linked with circadian clock disorders.

## Materials and Methods

### Ethics statement

Zebrafish handling procedures were approved by the Institute of Neuroscience, Shanghai Institutes for Biological Sciences, Chinese Academy of Sciences.

### Animals

Wild type AB strain zebrafish (*Danio rerio*) were obtained from National Zebrafish Resources of China (Shanghai Institutes for Biological Sciences). Adult zebrafish (6 months old, male) and larval zebrafish were maintained at 28°C under a 14h: 10h light/dark cycle. The light provided by three full spectrum fluorescent bulbs (13W, SHLY) was turned on at 9:00, and turned off at 23:00. The illuminance during light exposure was approximately 1400 lux, measured by a digital luxmeter (Model ZDS-10, SHXL) at the water surface. The fish were fed live brine shrimp at 10:00 and 15:30 every day.

### Analysis of zebrafish metabolic network

We downloaded all enzymatic reactions and their associated genes, i.e. Gene-Protein-Reaction (GPR) association, for zebrafish included in the KEGG database. We obtained the major metabolites participating in these reactions and the direction of reactions by parsing the KEGG pathway maps. The information of the zebrafish metabolic network can be found in S1 and S4 Datasets. The reactions are considered to be connected if they share the same substrate or product. This leads to an un-directed graph with reactions as nodes. The shortest distances between reactions on the graph were computed using Floyd’s algorithm implemented by allShortestPaths function of e1071 library in R program. The distances between genes coding for the enzymes catalyzing the reactions were computed as the sum of the shortest distances between their associated reactions (if they are associated with the same reaction) and the contribution from their phase difference in circadian gene expression. The contribution to the distance from phase difference is proportional to $4\sin^{2}\left(\frac{\Delta \theta }{2}\right)$, the squared distance between two unit vectors on the complex plane with phase angle difference Δ*θ*. The phase angle of the unit vector is related to the peak time of circadian oscillating gene between 0 and 24 by $\theta =\frac{p\times 2\pi }{24}$. The term $4\sin^{2}\left(\frac{p_{i}-p_{j}}{24}\right)\pi$ gives rise to the equivalent distance of 0, 1, 2, 3, 4 when the peak times of two genes differ by 0h, 4h, 6h, 8h, 12h respectively. Based on the combined distances in metabolic network and peak time difference, the gene tree was constructed by hierarchical clustering with complete linkage in R program. The tree structures in Fig. 1 and S1 Fig. were visualized using iTOL [42,43], and were not drawn to scale.

### Adult fish brain collection for microarray

To examine genome-wide circadian gene expression, we sampled adult male zebrafish whole brains in both LD (14h:10h light/dark) and DD conditions for microarray. In the LD group, 24 fish were placed in an adult zebrafish activity recording system (S1 Text). After 3 days 14h:10h LD acclimation, the fish were sacrificed and dissected at 4h intervals starting at ZT0 of the fourth LD cycle for 12 time points. In the DD group, 24 fish were also acclimated to 14h:10h LD cycle for 3 days, and then they were sacrificed every 4h starting at CT0 of the fourth day in DD condition for 12 time points. 2 fish were sampled individually in each time point. Every fish was killed by rapid decapitation, and the whole brain was removed and frozen immediately in liquid nitrogen and stored at -80°C. The collection of samples under darkness was performed under dim red light. Locomotor activity of each zebrafish was recorded during the experiment.

### Microarray data analysis

Microarray data analysis was performed as described before [38] with slight modifications (S1 Text). Briefly, the selection criteria used here were as follows: g-test p values less than 0.3 in both LD and DD with a dominant period set as 24 h. These cutoffs corresponded to an overall FDR less than 5% as computed from random permutation. The microarray data have been deposited in Gene Expression Omnibus (GEO) under accession number: GSE51279.

### Real-time PCR

Real-time PCR was performed as previously described [38]. The primer sequences of genes tested are listed in S3 Table.

### Morpholino injection

Morpholinos (MOs) were purchased from Gene Tools. Each MO except standard controls was designed to target the start codon region of the gene. The sequence of the *clock* MO was 5’-CAT CCC GGT CTA TGC TGG AGG TCA T-3’ as previously used by Li et al. [63]. The sequence for *impdh1a* MO was 5’-GAT CAG GTA ATC AGC CAT GAG TCT C-3’; *impdh1b* MO, 5’-CTC CGC TTA TCA GAT AGT CTG CCA T-3’; *impdh2* MO, 5’-GCT GAT TAA ATA GTC CGC CAT AGT-3’; and the standard control MO, 5’-CCT CTT ACC TCA GTT ACA ATT TAT A-3’. The sequences of *impdh1a* MO and *impdh2* MO were the same as those used previously [51]. MOs were used at the following doses: *clock* MO: 2.5ng; *impdh1a* MO, 8 ng; *impdh1b* MO, 9.6 ng; *impdh2* MO, 5.6 ng; standard control MO for *clock* knock-down, 2.5ng; standard control MO for three *impdhs* knock-down, 9.6ng. MOs were pressure-injected into 1- to 2-cell stage embryos at a volume of 1 nl using Picospritzer II injectors as described [64].

### Drug treatment

Mycophenolic acid (MPA, Sigma) was dissolved in dimethylsulfoxide (DMSO) at a stock concentration of 100mM and guanosine (Sigma) was also dissolved in DMSO at a stock concentration of 1M. At 4 dpf, WT larvae raised on a 14h: 10h LD cycle at 28°C were distributed into 35mm dishes with each dish holding 25 larvae in 5ml E3 buffer. Larvae were then exposed to drugs beginning at ZT0 (96 hpf) by directly pipetting MPA and/or guanosine stock solutions into each dish at the following final concentrations: 1μM, 10μΜ, 100μM; MPA and 100μM guanosine. The DMSO final concentration was adjusted to 1% in each dish. At their final concentrations, MPA and guanosine had no detectable effect on larval zebrafish behavior. Drug treated larvae were collected at 4h intervals starting at ZT0 of 5 dpf for 6 time points in LD conditions.

### BrdU labeling and confocal imaging

5 dpf larvae were incubated in 15% DMSO and 85% hanks (Gibco) solution with 10 mM bromodeoxyuridine (BrdU, Sigma) for 20min on ice. Then larvae were fixed in 4% paraformaldehyde (PFA) at 4°C overnight. After gradual dehydration and rehydration in methanol, larvae were permeabilized 10 min with 100% acetone prechilled to −20°C, and then 20 min with 0.1% trisodium citrate(with 0.1% Triton X-100) at RT. Larvae were rinsed three times with ddH_2_O and incubated in 2N HCl for 1h at RT. Subsequently larvae were washed in PBST (0.1% Triton X-100 in PBS) several times and blocked in 2% goat serum for 2h at RT. Then whole-mount immunofluorescence was performed with 1:10 anti-BrdU antibody (Roche) at 4°C overnight followed by incubation with secondary antibodies conjugated to fluorescein (1:1000) according to standard protocols. Brdu labeled larvae were embedded in 1.0% low-melting point agarose for imaging. Images were performed on an Olympus FV1000-MPE laser scanning confocal microscope. The number of Brdu positive nuclei was counted over 11 somites of the fish trunk between the anus and head.

### Melanocyte area measurement

Melanocyte area measurement was performed as described before [38]. The covered area of melanocytes was measured in the head region from the pineal gland to the optic vesicles excluding the eyes.

### Histology

Zebrafish eye histological analysis was conducted on vibratome sections. 5 dpf larvae were collected and fixed overnight at 4°C in 4% paraformaldehyde (PFA). After washing several times in PBST (0.1% Triton X-100 in PBS), the whole larvae were embedded in 4% low-melting point agarose and 50-μm-thick sections were cut using Leica VT 1200S. Images were obtained on an Olympus microscope (1X71) mounted with a DP72 digital camera controlled by DP2-BSW software. To maximize comparability across different *impdh* morphants, only sections with an optic nerve visible in the eyes were used. 5 dpf larvae morphological images were taken using an Olympus microscope SZX16 equipped with a DP71 CCD camera controlled by DP Controller software. Larval zebrafish anterior-posterior eye length and body length (from the middle of the mouth to the tip of the tail) was measured on images using ImageJ 1.41 software. Images were processed using Adobe Photoshop CS2.

### Sample collection for RNA-seq analysis

To validate the functions of the three *impdh* genes, we collected *impdh* MO injected embryos for RNA-seq. WT larvae, control and *impdh* morphants were raised under LD conditions and sampled simultaneously at 32 hpf. Each group had at least 40 embryos. Total RNA was extracted from each sample using Trizol. The quantity and quality of the RNA samples were assessed with a Qbit (invitrogen) and an Agilent 2100 bioanalyzer. RNA-seq was performed by Partner Institute for Computational Biology Omics Core. mRNA-seq libraries were prepared using the TruSeq RNA Sample Preparation v2 Kit following the manufacturer's protocol (Illumina). Briefly, 1ug poly-A containing mRNA was purified using poly-T oligo attached magnetic beads and then fragmented. The cleaved RNA fragments were primed with random hexamers into first strand cDNA, and then the second-strand cDNA was synthesized. The ends of double-strand cDNA (ds cDNA) were repaired into blunt ends, after which an adenosine base was added to the 3’ ends of ds cDNA and adaptors with a single T base overhang on 3’ end were ligated. These adapter-modified cDNA fragments were amplified by PCR which was performed with a PCR primer cocktail that annealed to the ends of the adapters. The products were purified, then the concentration and size distribution of the libraries were determined on a Qbit and an Agilent Bioanalyzer. Libraries were loaded onto single flow cells at concentrations of 9 pM to generate cluster densities of 750,000–850,000/mm^2^ following Illumina's standard protocol using the Illumina cBot and TruSeq SR cluster kit V3-HS (cBot). The flow cells were sequenced as 51 single reads on an Illumina HiSeq 2000 using TruSeq SBS sequencing kit version 3 and HCS version 2.0 data collection software, respectively. Base calling was performed using Illumina's real time analysis (RTA). The row sequence reads were exported in FASTQ format.

### RNA-seq data analysis

Sequence reads from RNA-seq data were aligned to the *Danio rerio* genome (Zv9) using the bowtie2 program [65] and all read mapping was carried out using TopHat2 [66]. All expression values were estimated using Cufflinks 2.1.1 (http://cufflinks.cbcb.umd.edu) [67] with the same annotations and reference sequences as TopHat2. FPKM value was obtained for each predicted transcript. We excluded the low abundant transcripts with FPKM less than 1 in all samples. Differential gene expression between each pair of samples was characterized by the log2 ratio of their FPKM values. A gene was considered as significantly differentially expressed if the absolute value of log2 ratio of FPKM values was greater than 2. The RNA-seq data have also been deposited in GEO under accession: GSE51279.

### Sample collection and preparation for ^1^H-NMR

WT AB larvae were raised in 14h:10h LD condition and collected at 5dpf. The larvae were sampled at 6h-interval: ZT0, ZT6, ZT12, ZT18, ZT24, and every time point has three biological replicates. For each sample, 50 larvae were collected in tubes for homogenization (91-PCS-CK14, PeqLab) and rapidly frozen in liquid nitrogen. Considering ideal freezing and storage conditions for pH-sensitive samples [68], the frozen samples were freeze dried overnight. Ceramic beads (91-PCS-CK14, PeqLab) and 1000 μL of acetonitrile / H_2_O (1:1) were added and the samples were extracted for 25 minutes at 4°C using a neolab Intellimixer (u1, 99 rpm). The samples were centrifuged at 14,000 rpm for 10 minutes at 4°C and the supernatants were freeze dried. For ^1^H-NMR measurement, the extracts were dissolved in a mixture of buffer (1.5 M KH_2_PO_4_, 2 mM NaN_3_, 0.1% TSP in D_2_O, pH 7.1) and D_2_O (1:9).

### ^1^H-NMR analysis

Spectra were recorded on a Bruker Avance II spectrometer using a ^1^H-BBI double resonance probe (Bruker Biospin GmbH). 1D NOESY spectra were recorded with presaturation for water suppression and 256 scans at 300 K (26.9°C). A prescan delay of 4 s was used together with a mixing time of 10 ms. Pulse lengths were determined automatically by the Bruker AU program *pulsecal*. 64k complex data points corresponding to a sweep width of 12,345.68 Hz were recorded.

### Processing of ^1^H-NMR data

All spectra were treated identically using an exponential apodization function, introducing an additional linewidth of 0.3 Hz and automated phasing, baseline correction and referencing using the Bruker macro *apk0*.*noe*. Preliminary peak assignment was done using databases (Chenomx (Chenomx Inc.), BBIOREFCODE (Bruker BioSpin GmbH), The Human Metabolome Database (HMDB)) and confirmed by spiking pure substances in the samples. Integration was done using AMIX (Bruker Biospin GmbH). Spectra were scaled to total intensity. Circadian oscillations were identified by fitting the mean intensities across 24 hours to a cosine function with periods between 20–28 and shifting phases. Statistical significance of differences between groups collected at different time points were assessed by ANOVA.

## Supporting Information

S1 Text Supplementary Materials and Methods for adult zebrafish activity recording and microarray analysis. (DOCX)

S1 Fig The rest of smaller clusters in the clustering of ZCOGs on zebrafish metabolic network. Four smaller isolated clusters (labeled as A, B, C, D) with size larger than 10 shown in the same manner as the main cluster in Fig. 1.
(TIF)

S2 Fig Adult zebrafish locomotor activity. (A) An infrared behavioral monitoring platform. The locomotor activities of all 24 fish can be tracked simultaneously. The activity curve of a selected fish in the red rectangle was displayed in real time. The color of the curve reflected the value of the moving: white, lower than freezing threshold; red, higher than burst threshold; green, between freezing threshold and burst threshold. Freezing threshold and burst threshold parameters for detection were matched to visual observation of the locomotion of individual fish. Locomotor activities of adult zebrafish under 5LD condition (B) and 3LD-2DD condition (C). Diamonds represent the 4h interval time points when the fish were collected for microarray analysis. The-y axis indicates the average value of pixels per second. The x-axis indicates light (white) and dark (black) in LD, subjective day (grey) and subjective night (black) in DD.
(TIF)

S3 Fig Adult fish brains collection for microarray. The locomotor activity of each adult fish was recorded before being sacrificed for microarray at 4h intervals in both LD and DD conditions. Every time point was generated using two independent fish. * In DD, fish 1 in CT12 escaped from its cell between CT8 and CT12, the recording data was missing during that period. This fish was sacrificed at CT12.
(TIF)

S4 Fig The phenotypes of WT, control morphant, and three *impdh* morphants at early stages of development. (A) *impdh1b* morphant development was faster than WT or control larvae while the *impdh2* morphant grew slower at 32 hpf and 52 hpf. (B) The body lengths were calculated in 5 dpf larvae. *impdh1b* knock-down promotes larval zebrafish development significantly. Error bars represent the standard error of mean (SEM) among independent replicates. * p<0.05; ***P<0.001, unpaired two-tailed Student’s t-test, scale bars, 500μm.
(TIF)

S5 Fig Genome-wide effects of three *impdh* homolog-specific knock-downs. (A) Heatmap shows the gene expression of WT & control and three *impdh* homolog-specific knock-downs. WT & control represents the combined mean gene expression of WT and control. (B) Venn diagram shows the overlapping genes affected by the three *impdh* homolog-specific knock-downs.
(TIF)

S6 Fig The expression of the three *impdh* homologs’ target genes. The differential expression of selected genes affected by the three *impdh* homologs knock-down: *dhfr* (A), *bcat1* (B), *rpe65a* (C) and *psat1* (D) have been validated by real-time PCR. Independent batches of samples from the ones used in RNA-seq were used for detection. Error bars represent the standard error of mean (SEM) among independent replicates. *** p<0.001, unpaired two-tailed Student’s t-test.
(TIF)

S7 Fig Circadian genes in the de novo purine pathway show different expression in larval and adult tissues. *atic* (A), *gart* (B), *pfas* (C), *ppat* (D) are widely rhythmically expressed in adult fish tissues. Error bars represent the standard error of mean (SEM) among independent replicates. *** p<0.001, unpaired two-tailed Student’s t-test.
(TIF)

S8 Fig Sequence alignment of Impdh homologs in zebrafish. IMPDH homologs are high conserved in different species. Zebrafish Impdh1a shows 90% identity and Impdh1b 91% identity with human IMPDH1. Zebrafish Impdh2 shares 91% identity with human IMPDH2.
(TIF)

S1 Table The water-soluble metabolites quantified by NMR in 5dpf larval zebrafish. (XLSX)

S2 Table ZCOGs that are linked to circadian metabolites. (XLSX)

S3 Table Primers used for real-time PCR. (XLS)

S1 Dataset The information of the zebrafish metabolic network. (XLSX)

S2 Dataset 283 ZCOGs are shared between adult and larval ZCOGs. (XLSX)

S3 Dataset Functional enrichment of the genes affected by *impdh*-specific. knock-downs. (XLSX)

S4 Dataset The circadian phases of the larval zebrafish metabolic network. (XLSX)
