# Supplementary material for: Integrative Analysis of Circadian Transcriptome and Metabolic Network Reveals the Role of De Novo Purine Synthesis in Circadian Control of Cell Cycle
Source: PLoS Comput Biol. 2015 Feb 25;11(2):e1004086. doi: 10.1371/journal.pcbi.1004086 (PMC4340947; doi:10.1371/journal.pcbi.1004086)
Supplement: S1 Text — (DOCX) [file pcbi.1004086.s001.docx]

**Supplemental Information**

**Integrative Analysis of Circadian Transcriptome and Metabolic Network Reveals the Role of de novo Purine Synthesis in Circadian Control of Cell Cycle**

Ying Li^1, §^, Guang Li^1, §^, Benjamin Görling^3, 4^, Burkhard Luy^3, 4^, Jiulin Du^2^, Jun Yan^1*^

^1^CAS-MPG Partner Institute for Computational Biology,

^2^Institute of Neuroscience,

Shanghai Institutes of Biological Sciences, Chinese Academy of Sciences, 320 Yue Yang Road, Shanghai, 200031, China

^3^Institute of Organic Chemistry, Karlsruhe Institute of Technology (KIT),

Fritz-Haber-Weg 6, D-76131 Karlsruhe, Germany

^4^Institute for Biological Interfaces, Karlsruhe Institute of Technology, Hermann-von-Helmholtz-Platz 1, D-76344 Eggenstein-Leopoldshafen,

Karlsruhe, Germany

^§^ These authors contributed equally to this work

^*^ To whom correspondence should be addressed: Jun Yan, email: [junyan@picb.ac.cn](mailto:junyan@picb.ac.cn)

**Running Title:** Circadian purine synthesis and cell cycle

**Keywords**: cell cycle/ circadian rhythm/ inosine 5'-phosphate dehydrogenase/ metabolism/ purine synthesis

**Supplementary** **Materials and Methods**

**Adult zebrafish activity recording**

Adult zebrafish were maintained in a recirculation fish housing system (ESEN). The transparent aquarium (60cm long×40cm wide×20cm deep) contained 24L water and had 24 individual cells (8cm long×8cm wide×15cm deep) separated by black plastic plates. The fish placed in each cell cannot see and interrupt each other. Water from recording aquarium flowed to a reservoir and then recirculated through biological and mechanical filters at a rate of 1200ml/min. Water conductivity was maintained between 450μS-550μS, and pH was maintained between 7.0-8.0. Heater (200W, EHEIM 3607, EHEIM), air diffuser (EHEIM 400, EHEIM) and pump (ESEN) were also provided in the reservoir. Locomotor activity of adult zebrafish was monitored by an automatic video-tracking system (ViewPoint S.A.). This system consisted of an infrared backlight emitter (HIR333C/H0, 850nm wavelength, ViewPoint S.A.) placed under the aquarium, an infrared camera (640 pixels×480 pixels, 25Fps, 1/3”CS M, 1.4 aperture, 2.8-12mm adapted lens, Point Grey Research Inc.) placed above all 24 cells and an associated software (ZebraLab3.10, ViewPoint S.A.). Images (25 frames/s) and videos were recorded through backlit chambers using an infrared camera. The movement of each zebrafish under light and dark condition can be recorded using videotrack quantization mode by which each image was converted to a digital value according to its luminosity. The data were further analyzed using custom R software.

**Microarray**

Total RNA of individual zebrafish brain was extracted using Trizol (Invitrogen) according to the manufacturer’s instruction. The quantity and quality of the RNA samples were assessed with a NanoDrop ND-1000 spectrometer (NanoDrop Technologies) and an Agilent 2100 bioanalyzer (Agilent Technologies). 12 LD RNA samples and 12 DD RNA samples were got for microarray by mixing the total RNA of 2 samples in the same Circadian Time with equal amounts. Microarrays were manufactured by Agilent Technologies, each contains 43603 probes for a whole-genome transcriptional profile. Purified total RNA of each sample was amplified and labeled with a fluorescent dye Cyanine 3 (Cy3) using a low-RNA input linear amplification kit following the manufacturer's protocol (5184-3523, Agilent Technologies). Cy3-labeled cRNA (800ng each) were hybridized to a zebrafish oligo microarray (G5219F, Agilent Technologies) for 17 h at 65°C. The hybridized microarrays were then washed according to the manufacturer's protocol. Microarray results were extracted using Agilent G2565BA Scanner and Feature Extraction software (v10.5.1, Agilent Technologies), subsequently analyzed by Gene-Spring software (v11.0.1, Agilent Technologies).

**Supplementary Figures**

**S1 Figure. The rest of smaller clusters in the clustering of ZCOGs on zebrafish metabolic network**

Four smaller isolated clusters (labeled as A, B, C, D) with size larger than 10 shown in the same manner as the main cluster in Fig 1.

**S2 Figure. Adult zebrafish locomotor activity**

(A) An infrared behavioral monitoring platform. The locomotor activities of all 24 fish can be tracked simultaneously. The activity curve of a selected fish in the red rectangle was displayed in real time. The color of the curve reflected the value of the moving: white, lower than freezing threshold; red, higher than burst threshold; green, between freezing threshold and burst threshold. Freezing threshold and burst threshold parameters for detection were matched to visual observation of the locomotion of individual fish.

Locomotor activities of adult zebrafish under 5LD condition (B) and 3LD-2DD condition (C). Diamonds represent the 4h interval time points when the fish were collected for microarray analysis. The-y axis indicates the average value of pixels per second. The x-axis indicates light (white) and dark (black) in LD, subjective day (grey) and subjective night (black) in DD.

**S3 Figure. Adult fish brains collection for microarray**

The locomotor activity of each adult fish was recorded before being sacrificed for microarray at 4h intervals in both LD and DD conditions. Every time point was generated using two independent fish. * In DD, fish 1 in CT12 escaped from its cell between CT8 and CT12, the recording data was missing during that period. This fish was sacrificed at CT12.

**S4 Figure. The phenotypes of WT, control morphant, and three *impdh* morphants at early stages of development**

(A) *impdh1b* morphant development was faster than WT or control larvae while the *impdh2* morphant grew slower at 32 hpf and 52 hpf. (B) The body lengths were calculated in 5 dpf larvae. *impdh1b* knock-down promotes larval zebrafish development significantly. Error bars represent the standard error of mean (SEM) among independent replicates. * p<0.05; ***P<0.001, unpaired two-tailed Student’s t-test, scale bars, 500μm.

**S5 Figure. Genome-wide effects of three *impdh* homolog-specific knock-downs**

(A) Heatmap shows the gene expression of WT & control and three *impdh* homolog-specific knock-downs. WT & control represents the combined mean gene expression of WT and control. (B) Venn diagram shows the overlapping genes affected by the three *impdh* homolog-specific knock-downs.

**S6 Figure. The expression of the three *impdh* homologs’ target genes**

The differential expression of selected genes affected by the three *impdh* homologs knock-down: *dhfr* (A), *bcat1* (B), *rpe65a* (C) and *psat1* (D) have been validated by real-time PCR. Independent batches of samples from the ones used in RNA-seq were used for detection. Error bars represent the standard error of mean (SEM) among independent replicates. *** p<0.001, unpaired two-tailed Student’s t-test.

**S7 Figure. Circadian genes in the de novo purine pathway show different expression in larval and adult tissues**

*atic* (A), *gart* (B), *pfas* (C), *ppat* (D) are widely rhythmically expressed in adult fish tissues. Error bars represent the standard error of mean (SEM) among independent replicates. *** p<0.001, unpaired two-tailed Student’s t-test.

**S8 Figure. Sequence alignment of Impdh homologs in zebrafish**

IMPDH homologs are high conserved in different species. Zebrafish Impdh1a shows 90% identity and Impdh1b 91% identity with human IMPDH1. Zebrafish Impdh2 shares 91% identity with human IMPDH2.

**Supplementary Tables**

**S1 Table. The water-soluble metabolites quantified by NMR in 5dpf larval zebrafish**

**S2 Table. ZCOGs that are linked to circadian metabolites**

**S3 Table. Primers used for real-time PCR**

**Supplementary Datasets**

**S1 Data. The information of the zebrafish metabolic network**

**S2 Data. 283 ZCOGs are shared between adult and larval ZCOGs**

**S3 Data. Functional enrichment of the genes affected by *impdh*-specific knock-downs**

**S4 Data. The circadian phases of the larval zebrafish metabolic network**
