# Supplementary figures and images for: Integrative Analysis of Circadian Transcriptome and Metabolic Network Reveals the Role of De Novo Purine Synthesis in Circadian Control of Cell Cycle
Source: PLoS Comput Biol. 2015 Feb 25;11(2):e1004086. doi: 10.1371/journal.pcbi.1004086 (PMC4340947; doi:10.1371/journal.pcbi.1004086)

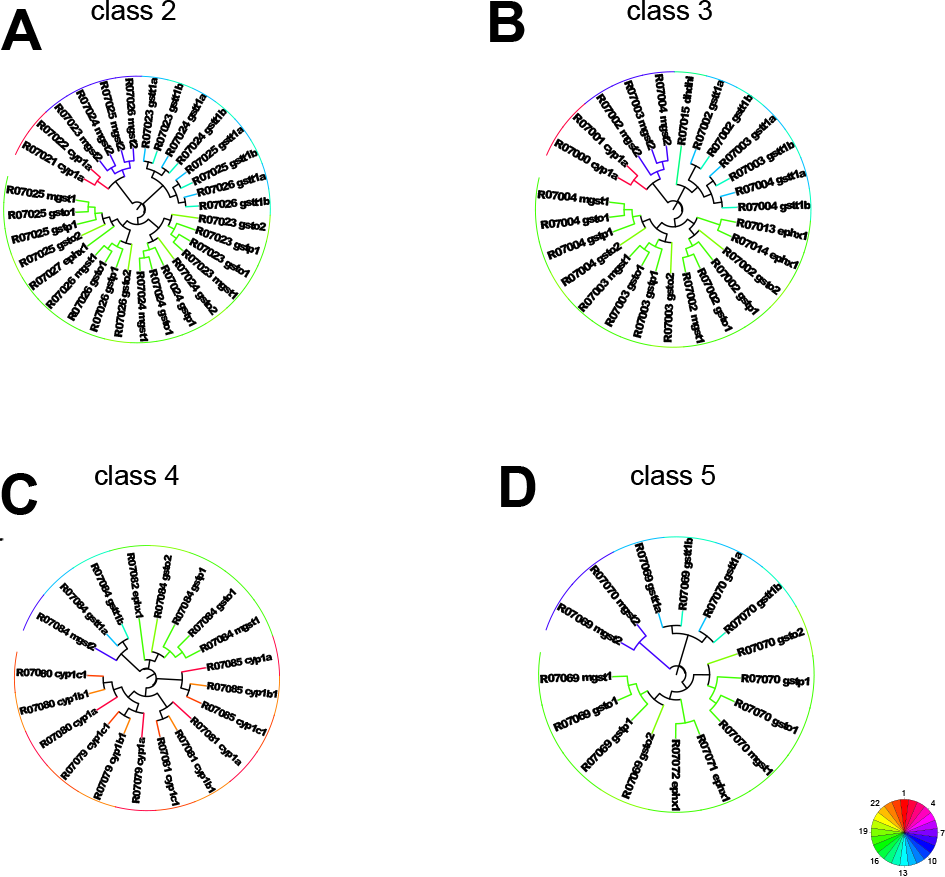

Supplement: S1 Fig — Four smaller isolated clusters (labeled as A, B, C, D) with size larger than 10 shown in the same manner as the main cluster in Fig. 1. (TIF) [file pcbi.1004086.s002.tif]

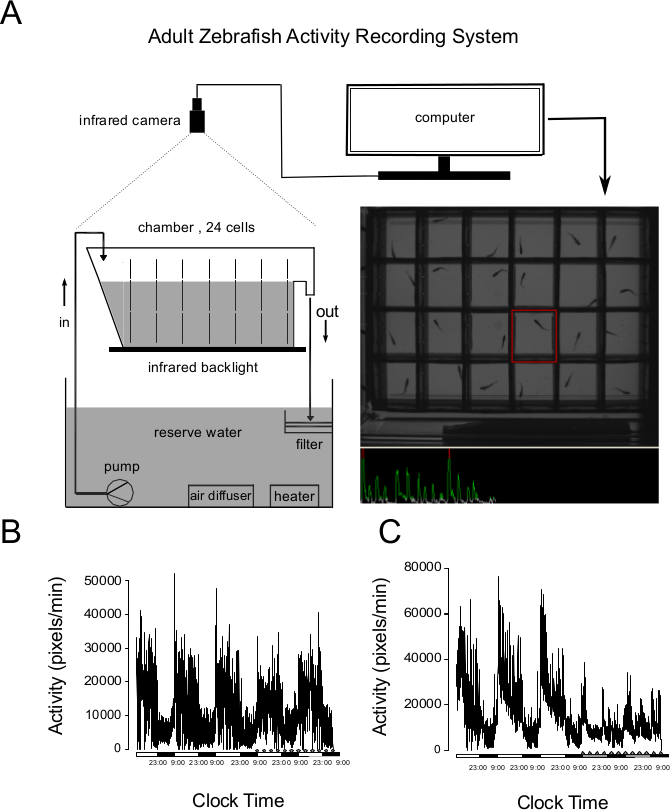

Supplement: S2 Fig — (A) An infrared behavioral monitoring platform. The locomotor activities of all 24 fish can be tracked simultaneously. The activity curve of a selected fish in the red rectangle was displayed in real time. The color of the curve reflected the value of the moving: white, lower than freezing threshold; red, higher than burst threshold; green, between freezing threshold and burst threshold. Freezing threshold and burst threshold parameters for detection were matched to visual observation of the locomotion of individual fish. Locomotor activities of adult zebrafish under 5LD condition (B) and 3LD-2DD condition (C). Diamonds represent the 4h interval time points when the fish were collected for microarray analysis. The-y axis indicates the average value of pixels per second. The x-axis indicates light (white) and dark (black) in LD, subjective day (grey) and subjective night (black) in DD. (TIF) [file pcbi.1004086.s003.tif]

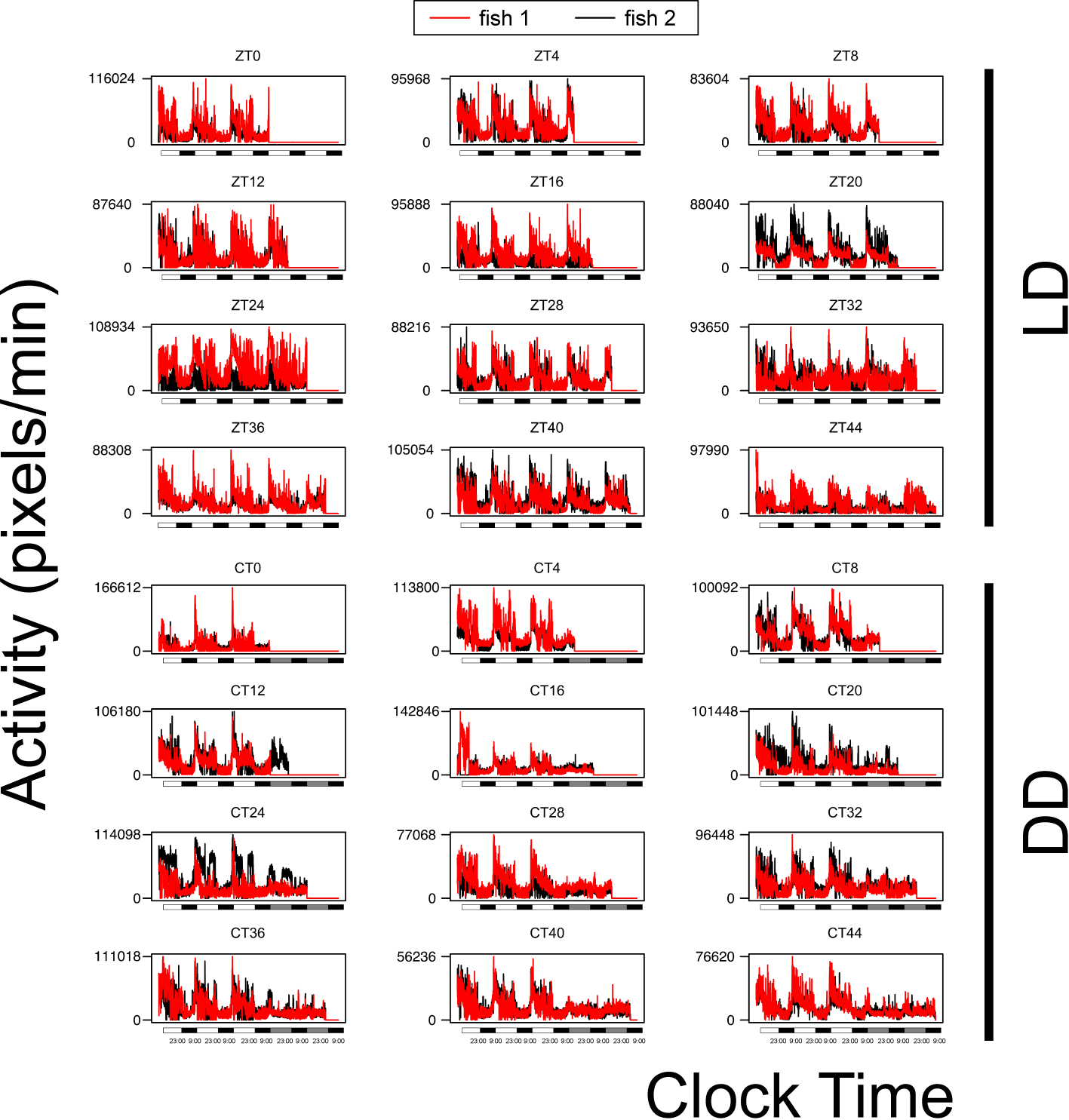

Supplement: S3 Fig — The locomotor activity of each adult fish was recorded before being sacrificed for microarray at 4h intervals in both LD and DD conditions. Every time point was generated using two independent fish. * In DD, fish 1 in CT12 escaped from its cell between CT8 and CT12, the recording data was missing during that period. This fish was sacrificed at CT12. (TIF) [file pcbi.1004086.s004.tif]

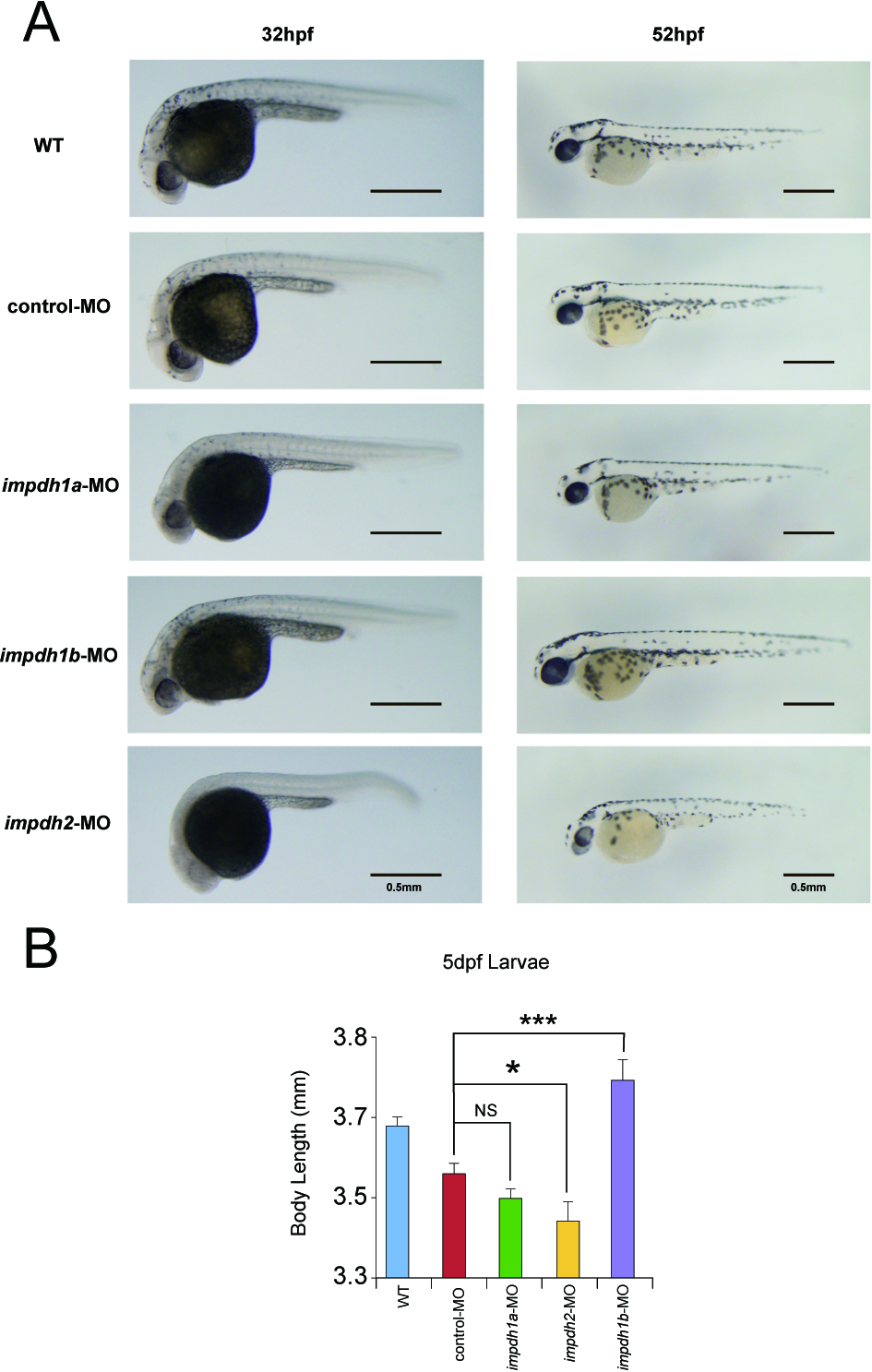

Supplement: S4 Fig — (A) impdh1b morphant development was faster than WT or control larvae while the impdh2 morphant grew slower at 32 hpf and 52 hpf. (B) The body lengths were calculated in 5 dpf larvae. impdh1b knock-down promotes larval zebrafish development significantly. Error bars represent the standard error of mean (SEM) among independent replicates. * p<0.05; ***P<0.001, unpaired two-tailed Student’s t-test, scale bars, 500μm. (TIF) [file pcbi.1004086.s005.tif]

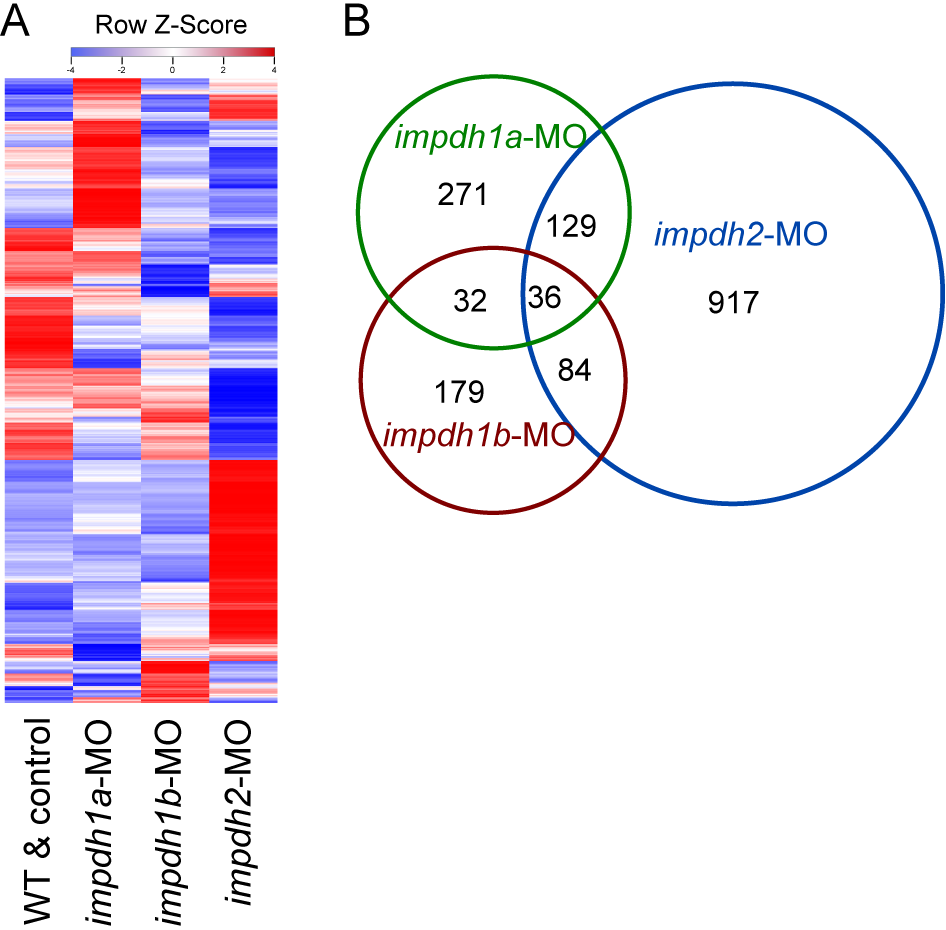

Supplement: S5 Fig — (A) Heatmap shows the gene expression of WT & control and three impdh homolog-specific knock-downs. WT & control represents the combined mean gene expression of WT and control. (B) Venn diagram shows the overlapping genes affected by the three impdh homolog-specific knock-downs. (TIF) [file pcbi.1004086.s006.tif]

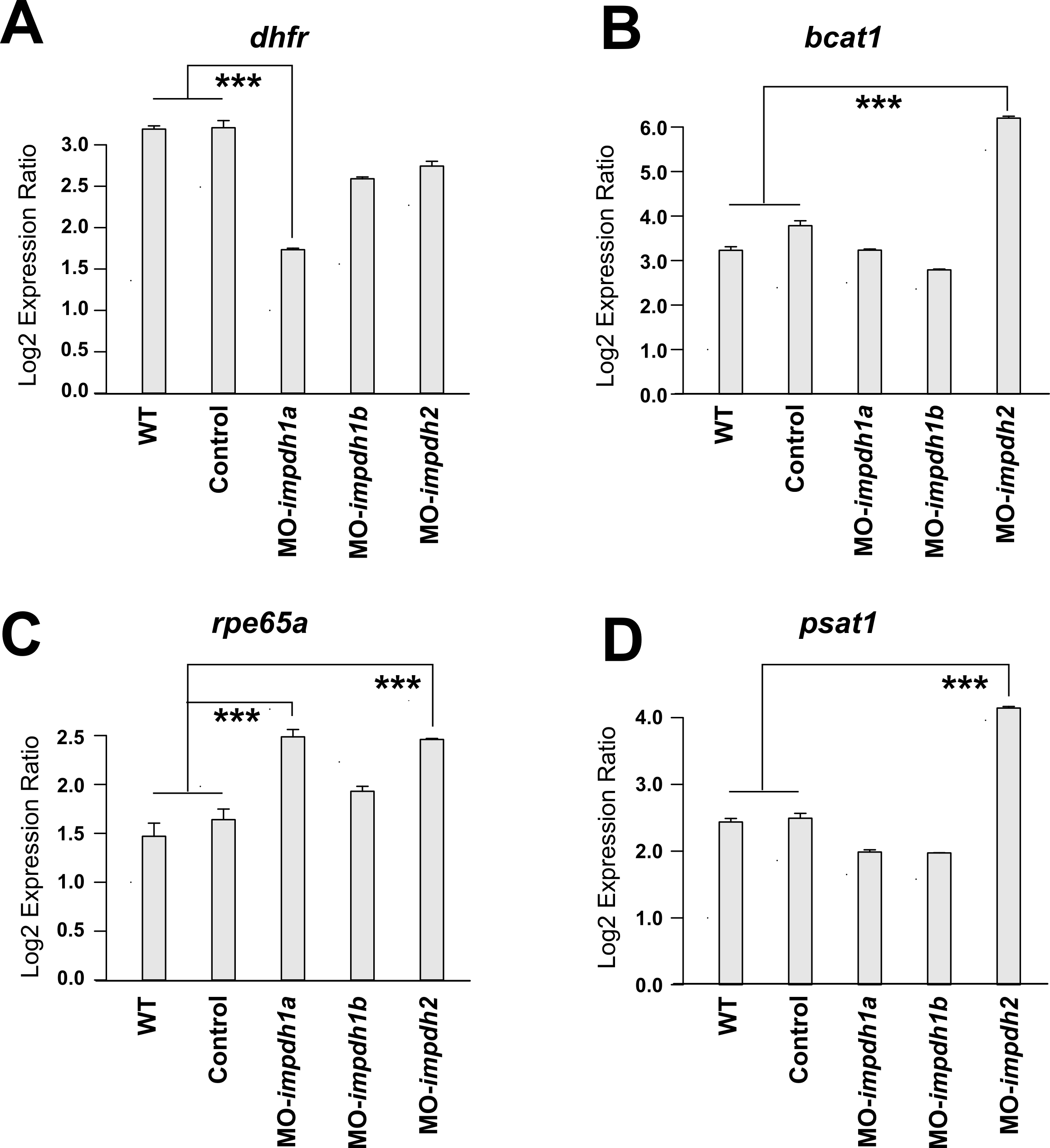

Supplement: S6 Fig — The differential expression of selected genes affected by the three impdh homologs knock-down: dhfr (A), bcat1 (B), rpe65a (C) and psat1 (D) have been validated by real-time PCR. Independent batches of samples from the ones used in RNA-seq were used for detection. Error bars represent the standard error of mean (SEM) among independent replicates. *** p<0.001, unpaired two-tailed Student’s t-test. (TIF) [file pcbi.1004086.s007.tif]

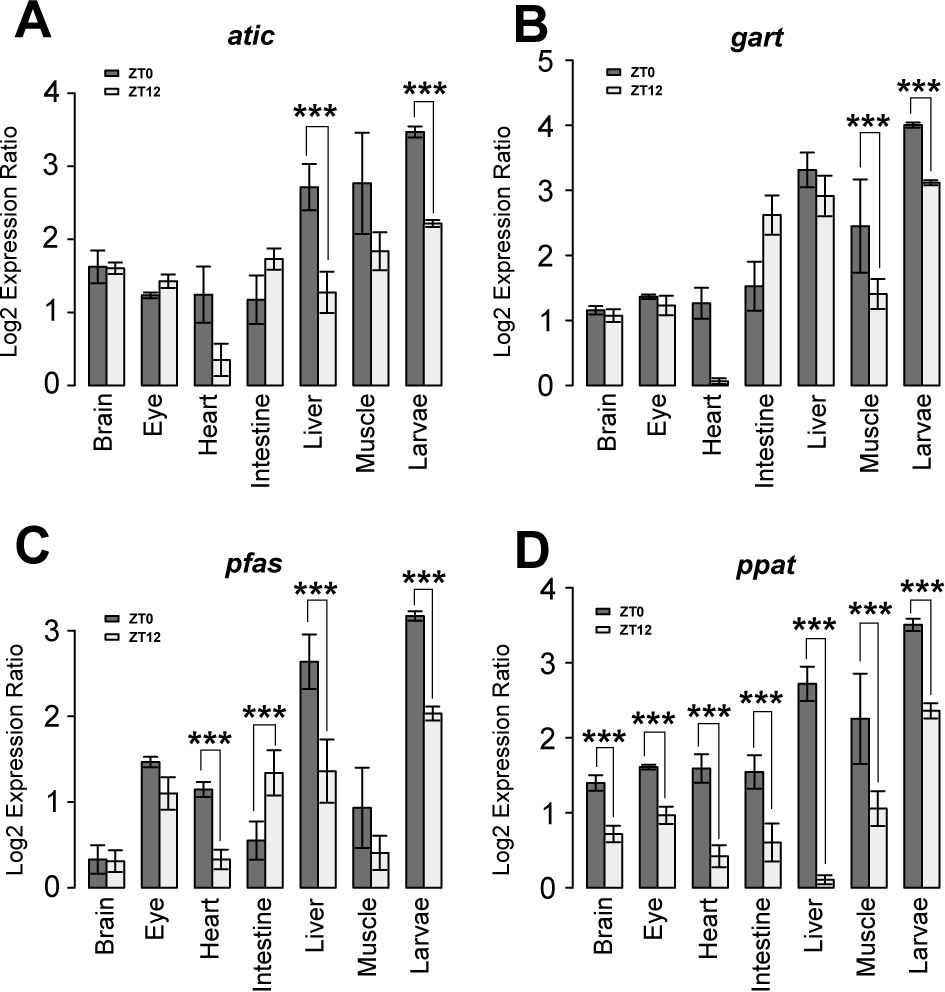

Supplement: S7 Fig — atic (A), gart (B), pfas (C), ppat (D) are widely rhythmically expressed in adult fish tissues. Error bars represent the standard error of mean (SEM) among independent replicates. *** p<0.001, unpaired two-tailed Student’s t-test. (TIF) [file pcbi.1004086.s008.tif]

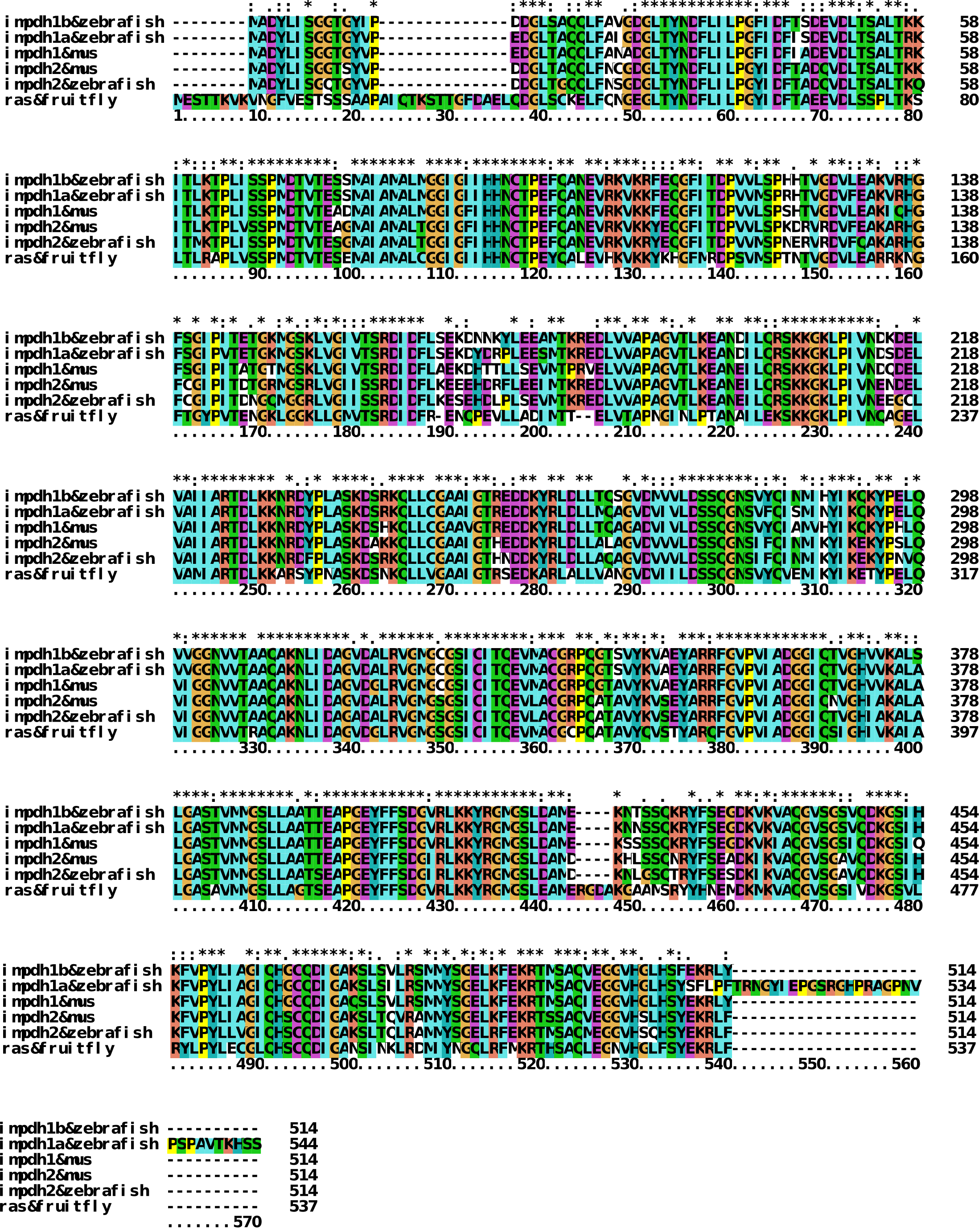

Supplement: S8 Fig — IMPDH homologs are high conserved in different species. Zebrafish Impdh1a shows 90% identity and Impdh1b 91% identity with human IMPDH1. Zebrafish Impdh2 shares 91% identity with human IMPDH2. (TIF) [file pcbi.1004086.s009.tif]
